# Neuropsychiatric symptoms in Vascular Cognitive Impairment: a systematic review

**DOI:** 10.1590/1980-57642015DN93000004

**Published:** 2015

**Authors:** Chan Tiel, Felipe Kenji Sudo, Gilberto Sousa Alves, Letice Ericeira-Valente, Denise Madeira Moreira, Jerson Laks, Eliasz Engelhardt

**Affiliations:** 1 Instituto de Neurologia Deolindo Couto, Setor de Neurologia Cognitiva e do Comportamento-INDC-CDA/IPUB, UFRJ, Rio de Janeiro RJ, Brazil.; 2 Instituto de Psiquiatria, Universidade Federal do Rio de Janeiro (UFRJ), Rio de Janeiro RJ, Brazil.; 3 Departamento de Medicina Clínica, Universidade Federal do Ceará, Fortaleza CE, Brazil.; 4 Serviço de Radiologia, Instituto de Neurologia Deolindo Couto (UFRJ); Hospital Pró-Cardíaco, Rio de Janeiro RJ, Brazil.; 5 Universidade do Estado do Rio de Janeiro, Rio de Janeiro RJ, Brazil.

**Keywords:** neuropsychiatric symptoms, BPSD, NPI, Vascular Cognitive Impairment, Vascular Dementia, Alzheimer's disease

## Abstract

Neuropsychiatric symptoms or Behavioral and Psychological Symptoms of Dementia (BPSD) are common and invariably appear at some point during the course of the disease, mediated both by cerebrovascular disease and neurodegenerative processes. Few studies have compared the profiles of BPSD in Vascular Cognitive Impairment (VCI) of different subtypes (subcortical or cortical) and clinical stages (Vascular Cognitive Impairment No Dementia [VaCIND] and Vascular Dementia [VaD]).

**Objective:**

To review the BPSD associated with different subtypes and stages of VCI using the Neuropsychiatric Inventory (NPI).

**Methods:**

Medline, Scielo and Lilacs databases were searched for the period January 2000 to December 2014, with the key words: "BPSD AND Vascular Dementia, "NPI AND Vascular Dementia" and "NPI AND VCI. Qualitative analysis was performed on studies evaluating BPSD in VCI, using the Neuropsychiatric Inventory (NPI).

**Results:**

A total of 82 studies were retrieved of which 13 were eligible and thus included. Among the articles selected, 4 compared BPSD in Subcortical Vascular Dementia (SVaD) versus Cortical-Subcortical Vascular Dementia (CSVaD), 3 involved comparisons between SVaD and VaCIND, 1 study analyzed differences between CSVaD and VaCIND, while 5 studies assessed BPSD in CSVaD. Subcortical and Cortical-Subcortical VaD were associated predominantly with Apathy and Depression. VaCIND may present fewer behavioral symptoms than VaD.

**Conclusion:**

The profile of BPSD differs for different stages of VCI. Determining the most prevalent BPSD in VCI subtypes might be helpful for improving early diagnosis and management of these symptoms.

## Abstract (pt)

Sintomas Neuropsiquiátricos ou Sintomas de Comportamento e Psicológicos da Demência (SCPD) são comuns e aparecem em algum ponto no curso da enfermidade, mediado por doenças cerebrovasculares e processos neurodegenerativos. Poucos estudos compararam os perfis dos SCPD no Comprometimento Cognitivo Vascular (CCV) de diferentes subtipos (subcortical ou córtico-subcortical) e diferentes estágios clínicos (Comprometimento Cognitivo Vascular Não Demência [CCV-ND] e Demência Vascular [DV]).

**Objetivo:**

Revisar os SCPD associados aos diferentes subtipos e estágios do CCV, com o Inventário Neuropsiquiátrico (INP).

**Métodos:**

Medline, Scielo e Lilacs foram pesquisados de janeiro de 2.000 até dezembro de 2.014, com as palavras chaves: "BPSD AND Vascular Dementia", "NPI AND Vascular Dementia" e "NPI AND VCI". Uma análise quantitativa foi feita nos estudos que avaliaram sintomas comportamentais no CCV através do INP.

**Resultados:**

Um total de 82 estudos foram identificados e 13 estudos foram considerados elegíveis e incluídos. Destes artigos, 4 compararam SCPD entre DV Córtico-Subcortical (DVCS) e DV Subcortical (DVS), 3 artigos compararam DVS e CCVND, 1 artigo analisou diferenças entre DVCS e CCVND, e 5 estudos avaliaram os SCPD em DVCS. Tanto a DVCS quanto a DVS associaram-se principalmente a Apatia e Depressão. O CCVND pode apresentar menos sintomas comportamentais que a DV.

**Conclusão:**

O perfil dos SCPD difere entre diferentes estágios do CCV. O entendimento dos SCPD mais prevalentes nos subtipos do CCV poderia ser útil para facilitar o diagnóstico precoce e o manejo desses sintomas.

## INTRODUCTION

Vascular Dementia (VaD) is considered the second-most-common type of dementing illness, accounting for a significant proportion of total dementia cases.^1,2^ Vascular Cognitive Impairment (VCI) is a broader dimensional term,^3^ encompassing subjects with mild cognitive impairment and no incapacity on activities of daily living, referred to as Vascular Cognitive Impairment No Dementia (VaCIND), as well as conditions associated with significant cognitive impairment and decline in functional status (VaD).^4,5^ Clinical features can vary considerably depending on the extension and location of vascular lesions. Moreover, VCI may be associated with large-vessel disease, which often leads to extensive cortical-subcortical damage, or with insidious occlusion/semi-occlusion of small penetrating arterioles, resulting in subcortical vascular disease.^6,7^

Comparisons among signs and symptoms of different subtypes of VCI may help understand how different mechanisms of brain damage can produce convergent or divergent clinical features, which in turn may lead to a better comprehension of the pathophysiology of the disorder. Among these manifestations, Neuropsychiatric Symptoms are of great importance, considering that they almost invariably appear at some point of the natural course of VCI in addition to cognitive impairment.^8-12^ Also referred as Behavioral and Psychological Symptoms of Dementia (BPSD),^13^ these features are frequently associated with growing levels of distress both in persons with dementia and in their caregivers, as well as with higher risk for adverse outcomes and increased use of health care resources.^14^ Clinically, BPSD have a major impact on the patient's functional and cognitive status.^15^

Despite considerable advances in the detection of brain vascular-related syndromes in recent years, the association between vascular lesions and both cognitive symptoms and BPSD in VCI remains controversial. For instance, data in the literature suggests that BPSD may occur in VCI, regardless of the development of dementia.^16,17^ BPSD may also appear at any stage, induced by cerebrovascular lesions disrupting the cortical-subcortical circuits between prefrontal cortex connections to limbic nuclei of the basal ganglia and thalamus, and other limbic system structures.^18-20^ This suggests that better characterization of vascular-related BPSD, and the underlying mechanisms of brain injury associated with these features, is still needed so as to allow the adoption of effective evidence-based prophylactic and therapeutic measures.

Among methods for assessing BPSD, the Neuropsychiatric Inventory (NPI)^21^ is a valid and reliable instrument, originally developed to assess 10 neuropsychiatric disturbances commonly seen in dementia. Subsequently, it has been modified to evaluate 12 disturbances including agitation, aberrant motor behavior, anxiety, elation, irritability, depression, apathy, disinhibition, delusions, hallucinations, sleep and appetite changes.^22^ Currently, the NPI is the most frequently used instrument for evaluating these symptoms in research studies and clinical practice. It is a semistructured interview which rates the frequency and severity of BPSD for the month preceding assessment. The NPI has been translated and validated to Brazilian Portuguese.^23^

To date, few studies have compared neuropsychiatric disturbances among VCI subgroups (cortical or subcortical, "pure" or mixed etiology, VaD or VaCIND). Thus, the aim of the present review was to examine BPSD profiles in VCI subgroups, primarily addressing two issues:

[1] which are the most prevalent BPSD in VCI, and
[2] whether different clinical presentations of VCI present different BPSD profiles. It can be hypothesized that the BPSD in VCI have a heterogeneous pattern, depending on the clinical stage and mechanism of brain lesion.

## METHODS

**Search strategy and criteria.** To identify articles, a systematic search was performed in PUBMED/MedLine, Scielo and Lilacs databases for English-language articles published between January 2000 and December 2014, followed by a manual search of the reference lists of relevant articles retrieved. The results of three searches, which employed the following key words: "BPSD AND Vascular Dementia", "NPI AND Vascular Dementia" and "NPI AND VCI" were combined.

Studies were selected for inclusion in the present systematic review if they met all of the following criteria:

[a] studies that assessed patients diagnosed with VaD according to the 4^th^ edition of the Diagnostic and Statistical Manual of Mental Disorders (DSM-IV)^24^ criteria or the NINDS-AIREN criteria^1^ and/or studies that assessed Va-CIND subjects, diagnosed according to well-recognized criteria;^24,26^
[b] studies that assessed BPSD in VCI;
[c] studies that evaluated BPSD using the NPI;
[d] studies that presented data on the prevalence and/or incidence rates of BPSD. Studies involving Alzheimer's disease (AD) or mixed dementia (MD) subjects were included if within a comparison group for VCI subjects.

The following exclusion criteria were used:

[a] articles with AD samples only;
[b] studies that assessed dementia other than AD or VaD;
[c] clinical trials with medications;
[d] reviews, posters, case reports, essays or letters.

**Data extraction.** Data from those studies meeting the inclusion criteria were extracted by one of the authors (CT) and reviewed by other researchers from the group (JL, FKS). In cases of disagreement among the two, discussion included the whole group to reach a consensus. Information about the clinical diagnosis, sample size, assessment instrument for BPSD, and the prevalence of BPSD categories were drawn from the selected studies.

**Statistical analysis.** Data were analyzed using the IBM Statistical Package for Social Sciences version 20 for Windows. Initially, the Shapiro-Wilk test was performed to verify the normality of data (prevalence of BPSD). Since significance exceeded .05 for all variables, One-Way ANOVA was performed to compare statistically significant differences between BPSD prevalence in the SVaD (Subcortical Vascular Dementia), VaCIND (Vascular CIND) and CSVaD (Cortical-Subcortical Vascular Dementia) groups. Tukey's Post-hoc test was performed to identify groups with significantly different prevalences of BPSD. The level of significance was set at .05.

## RESULTS

**Figure 1:**
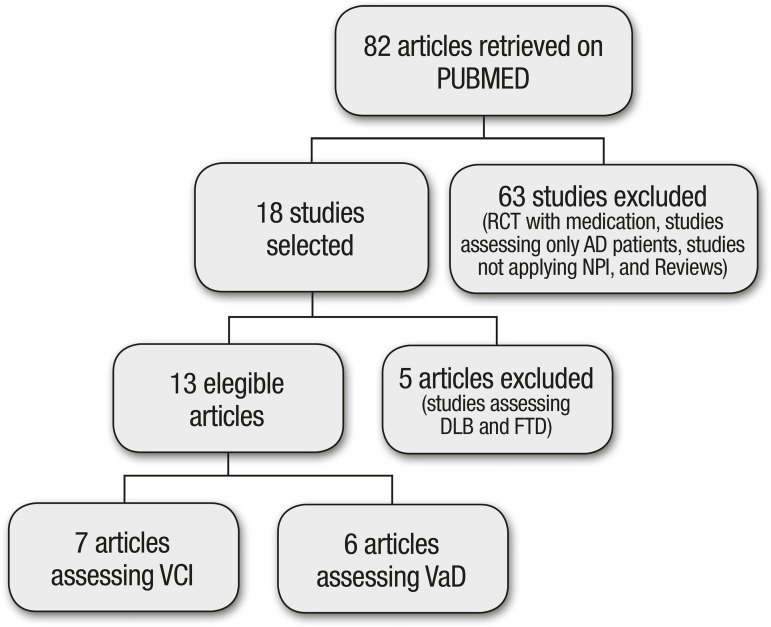
Flowchart of search strategy and article selection. RCT: Randomized clinical trial; AD: Alzheimer’s Disease; NPI: Neuropsychiatric Inventory; DLB: Dementia with Lewy Bodies; FTD: Frontotemporal dementia; VCI: Vascular Cognitive Impairment; VaD: Vascular Dementia.

Of the total 82 articles initially retrieved, 13 fulfilled the inclusion criteria and were included in this review. No studies were identified on the Lilacs and Scielo databases using the previously described search strategy. Figure 1 shows the stages of selection of papers included in this article.

The number of participants in the studies ranged from 51 to 938 patients. Diagnostic groups included different stages of VCI (VaCIND and VaD), etiologies (VCI, AD and MD) and subtypes of VaD (cortical and cortical-subcortical). Operational criteria for VCI were the DSM-IV,^24^ NINDS-AIREN,^1^ Hachinski's Ischemic Score,^27^ Clinical Dementia Rating,^28^ Fazekas scale,^29^ and the Canadian Study of Health and Ageing criteria for VaCIND.^30^ These data are summarized in Table 1.

**Table 1:** Characteristics of samples, diagnostic groups and operational criteria in the studies selected.

Year	Diagnosis	n	Diagnostic criteria	References
2007	V-CIND + VaD + MD (AD+VaD)	157	DSM-IV, CDR, HIS	Chiu et al.^17^
2010	VaD	484	DSM-IV, NINDS-AIREN	Staekenborg et al.^42^
2012	SVD + V-CIND	73	DSM-IV, Fazekas, CDR	Chin et al.^40^
2013	SVD + V-CIND	127	DSM-IV, Fazekas, CDR	Kim et al.^43^
2013	VaD + V-CIND	60	NINDS-AIREN, DSM-IV, HiS, CDR	Gupta et al.^31^
2013	VaD	51	NINDS-AIREN,DSM-IV	Pan et al.^44^
2014	VaD + V-CIND	60	NINDS-AIREN, CSHA	Gupta et al.^45^
2000	AD + VaD	329	DSM-IV, NINDS-AIREN, CDR	Lyketsos et al.^9^
2005	AD + VaD (cortical + subcortical)	536	DSM-IV, NINCDS-ADRDA, CDR	Fuh et al.^35^
2009	AD + VaD	154	DSM-IV, CDR, NINCDS-AIREN	Hsieh et al.^15^
2008	AD + VaD (cortical + subcortical)	65	DSM-IV, NINCDS-ADRDA, HIS	Fernández-Martínez et al.^32^
2012	AD + VaD	302	DSM-IV, NINCDS-ADRDA, NINDS-AIREN	D’onófrio et al.^46^
2014	AD + VaD (cortical + subcortical)	100	DSM-IV, NINDS-AIREN	Bandyopadhyay et al.^33^

AD: Alzheimer Dementia; VaD: Vascular Dementia; MD: Mixed Dementia; SVD: Subcortical Vascular Dementia; VCIND: Vascular cognitive Impairment-No Dementia; DSM-IV: Diagnostic and Statistical Manual of Mental Disorders 4^th^ edition; CDR: Clinical Dementia Rating; HIS: Hachinski ischaemic Score; Fazekas: Fazekas scale; CSHA: Canadian Study of Health and Ageing; NINDS-AIREN: National Institute of Neurological Disorders and Stroke and Association - Internationale pour la Recherché et lÉnseignement en Neurosciences; NINCDS-ADRDA: National Institute of Neurological and Communicative Disorders and Stroke and the Alzheimer’s Disease and Related Disorders Association.

BPSD were identified in all diagnostic groups at a high prevalence: 83.8 [96.4%] in SVaD, 59.7 [100%] in CSVaD and 47.5 [89%] in VaCIND. Apathy and Depression were the most frequent BPSD, followed by Irritability, Anxiety and Agitation. In another study, a higher prevalence of Euphoria was identified in VaCIND than in VaD (7.14% *vs* 3.13%).^31^ Agitation/Aggression symptoms appeared to be equally prevalent in CSVaD (21.4 [62.9%]) and in SVaD (22.7 [47.62%]). Also, patients with SVaD presented comparable Aberrant Motor Behavior (up to 38.10%) and Hallucinations (up to 28%) as patients with CSVaD (5-61.5% and 1-30.8%, respectively). Table 2 depicts the prevalences of BPSD and the NPI scores in the studies.

**Table 2:** Comparison of mean NPI subitem scores in studies assessing VCI subjects.

References (n)	Del	Hal	Agi	Dep	Anx	Eup	Apa	Dis	Irr	Ama	Sle	App	Total
**Subcortical Vascular Dementia (SVaD)**													
Fuh et al., 2005^35^ (n=161)	(--) [31.1%]	(--) [21.0%]	(--) [44.1%]	(--) [45.0%]	(--) [33.8%]	(--) [14.9%]	**(--)** **[47.2%]**	(--) [24.0%]	(--) [41.6%]	(--) [27.9%]	(--) [49.4%]	(--) [40.3%]	(--) [--]
Chiu et al., 2007^17^ (n=95)	1.7 (3.6) [26%]	1.6 (3.5) [28%]	2.1 (3.8) [32%]	2.8 (4.0) [49%]	1.9 (3.6) [32%]	0.4 (1.5) [6%]	4.6 (5.3) [48%]	0.9 (2.5) [16%]	2.9 (4.3) [41%]	2.3 (4.4) [26%]	5.2 (5.1) [60%]	2.5 (4.1) [31%]	27.7 (22.2) [92%]
Fernández-Martínes et al., 2008^32^ (n=22)	(--) [9.1%]	(--) [4.5%]	(--) [22.7%]	(--) [31.8%]	(--) [27.3%]	(--) [9.1%]	(--) [54.5%]	(--) [22.7%]	(--) 31.8%]	(--) [0.0]	**(--)** **[4.5%]**	**(--)** **[13.6%]**	11.82 (9.75) [96.4%]
Staekenborg et al., 2010^41^ (n=401)	0.4 (1.4) [12%]	0.1 (1.3) [7%]	1.1 (3.0) [38%]	1.4 (2.5) [45%]	1.1 (3.1) [34%]	0.1 (1.0) [5%]	3.8 (3.4) [67%]	0.5 (1.6) [16%]	1.2 (2.1) [41%]	0.6 (1.6) [15%]	1.1 (2.8) [33%]	1.4 (2.3) [29%]	--(--) [93%]
Chin et al., 2012^42^ (n=42)	0.31 (1.14)	0.33 (1.32)	1.40 (1.96)	1.33 (1.78)	1.43 (1.48)	0.31 (1.32)	3.86 (4.00)	1.00 (2.48)	2.10 (2.62)	2.26 (4.20)	2.21 (3.73)	2.40 (3.68)	18.95 (16.18)
Kim et al., 2013^43^ (n=68)	(--) [19.1%]	(--) [10.3%]	(--) [42.6%]	(--) [50.0%]	(--) [41.8%]	(--) [2.9%]	(--) [57.4%]	(--) [27.9%]	(--) [45.6%]	(--) [16.2%]	(--) [27.9%]	(--) [35.3%]	(--) [83.8%]
Gupta et al., 2014^45^ (n=21)	1.7 [33.33%]	0.1 [4.76%]	3.9 [47.62%]	6.0 [76.19%]	4.0 [57.14%]	0.0 [0.0%]	5.9 [61.90%]	0.4 [14.29%]	4.2 [52.38%]	2.8 [38.10%]	3.7 [52.38%]	4.3 [80.95%]	(--) [95%]
**Cortical-Subcortical Vascular Dementia (CSVaD)**													
Lyketsos et al., 2000^9^* (n=62)	**0.24 (0.98)** **[8.1%]**	0.29 (0.96) [12.9%]	1.10 (2.03) [32.2%]	**2.78 (10.80)** **[32.3%]**	0.57 (1.70) [17.7%]	0.10 (0.79) [1.6%]	0.43 (3.30) [22.2%]	0.52 (1.80) [11.3%]	0.41 (0.94) [17.7%]	0.19 (0.76) [8.1%]	--(--) [--]	--(--) [--]	7.70 (13.50) [59.7%]
Fuh et al., 2005^35^ (n=35)	(--) [31.4%]	(--) [25.7%]	(--) [62.9%]	(--) [51.0%]	(--) [51.0%]	(--) [11.0%]	**(--)** **[62.9%]**	(--) [34.3%]	(--) [51.4%]	(--) [26.5%]	**(--)** **[65.7%]**	(--) [45.7%]	(--) [--]
Fernández-Martínes et al., 2008^32^ * (n=6)	0.75 (2.44) [14.3%]	0.43 (2.27) [3.6%]	0.96 (2.27) [21.4%]	0.96 (1.86) [35.7%]	0.96 (1.64) [28.6%]	0.14 (0.59) [7.1%]	4.11 (3.85) [64.3%]	0.54 (1.29) [17.9%]	1.75 (2.95) [35.7%]	0.29 (1.05) [7.1%]	**0.21 (1.13)** **[3.6%]**	**0.71 (1.90)** **[14.3%]**	11.82 (9.75) [96.4%]
Hsieh et al, 2008^15^* (n=77)	2.46 (4.34) [26.9%]	1.46 (2.77) [30.8%]	1.73 (3.19) [30.8%]	3.30 (2.81) [65.4%]	1.23 (2.05) [34.6%]	2.00 (3.09) [34.6%]	5.04 (2.90) [65.4%]	0.00 (0.00) [0.0%]	0.92 (1.89) [23.1%]	3.31 (3.08) [61.5%]	**5.23 (4.07)** **[73.1%]**	0.00 (0.00) [0.0%]	26.69 (9.92) [100%]
Staekenborg et al., 2010^42^ (n=83)	0.3 (1.5) [7%]	0.0 (0.1) [1%]	1.5 (2.3) [48%]	1.1 (1.6) [43%]	1.5 (2.3) [39%]	0.2 (0.7) [13%]	2.1 (2.9) [54%]	0.4 (1.1) [16%]	1.7 (2.5) [49%]	0.2 (0.9) [5%]	1.0 (2.8) [31%]	1.5 (2.4) [27%]	(--) [--]
D’onófrio et al. 2012^46^* (n=136)	(--) [11.9%]	(--) [9.7%]	**(--)** **[33.6%]**	(--) [59.7%]	(--) [49.3%]	(--) [1.5%]	(--) [56.0%]	(--) [0.0%]	**(--)** **[38.8%]**	(--) [17.9%]	(--) [63.4%]	(--) [54.5%]	(--) [69.4%]
Gupta et al., 2013^31^* (n=32)	0.96 [21.88%]	0.09 [6.25%]	3.81 [53.13%]	6.53 [75.0%]	3.03 [40.63%]	0.28 [3.13%]	5.37 [59.38%]	0.5 [9.38]	4.18 [59.38%]	1.78 [28.13%]	3.28 [50.0%]	4.43 [84.38%]	(--) [100%]
Pan et al., 2013^44^* (n=51)	1.55 (0.69)	1.30 (0.51)	5.55 (0.90)	4.21 (0.81)	2.32 (0.81)	3.75 (0.65)	3.33 (0.71)	2.75 (0.68)	3.2 (0.92)	4.25 (0.69)	5.32 (0.83)	4.08 (0.57)	-- (--)
Gupta et al., 2014^45^ (n=25)	0.5 [16%]	0.2 [8%]	3.8 [52%]	6.1 [72%]	2.3 [36%]	0.7 [12%]	1.9 [28%]	0.6 [8%]	4.8 [72%]	0.9 [20%]	1.8 [32%]	3.5 [68%]	(--) [95%]
Bandyopadhyay et al., 2014^33^ * n=50)	(--) [8%]	(--) [12%]	**(--)** **[38%]**	**(--)** **[46%]**	**(--)** **[28%]**	(--) [6%]	**(--)** **[40%]**	(--) [10%]	**(--)** **[16%]**	**(--) ** **[28%]**	**(--) ** **[40%]**	**(--) ** **[28%]**	(--) [96%]
**Vascular CIND (VaCIND)**													
Chin et al., 2012^40^ (n=31)	0.00 (0.00)	0.00 (0.00)	0.55 (1.73)	0.26 (0.58)	0.39 (0.76)	0.03 (0.18)	1.00 (2.14)	0.23 (1.09)	0.52 (1.29)	0.52 (1.29)	0.19 (0.65)	0.10 (0.54)	3.26 (5.64)
Kim et al., 2013^43^ (n=59)	(--) [1.7%]	(--) [0.0%]	(--) [11.9%]	(--) [25.4%]	(--) [22.0%]	(--) [0.0%]	(--) [20.3%]	(--) [8.5%]	(--) [25.4%]	(--) [0.0%]	(--) [5.1%]	(--) [13.6%]	(--) [47.5%]
Chiu et al., 2007^17^ (n=41)	0.7 (1.8) [17%]	0.0 (0.2) [2%]	0.5 (1.6) [12%]	1.4 (2.5) [41%]	0.9 (1.9) [29%]	0.1 (0.3) [2%]	1.7 (3.7) [24%]	0.3 (1.9) [5%]	1.3 (2.6) [34%]	0.2 (1.3) [2%]	5.1 (5.4) [59%]	1.7 (3.4) [24%]	13.5 (11.6) [85%]
Gupta et al., 2013^31^ (n=28)	0.6 [17.86%]	0.07 [3.57%]	2.25 [25%]	4.6 [71.43%]	2.5 [42.86%]	0.5 [7.14%]	0.46 [7.14%]	0.25 [7.14%]	3.14 [42.86%]	0.96 [14.29%]	0.82 [14.29%]	2.1 [42.86%]	-- (--) [89%]

Values expressed as mean (standard deviation). VaD: Vascular dementia; CIND: Cognitive Impairment No-Dementia; Del: Delusions; Hal: Hallucinations; Agi: Agitation; Dep: Depression; Anx: Anxiety; Eup: Euphoria; Apa: Apathy; Dis: Disinhibition; Irr: Irritability; Ama: Aberrant motor activity; Sle: Sleep disturbance; App: Eating disorder.

* VaD diagnosed according to NINDS-AIREN criteria. Values in bold were significantly different from scores in AD subjects.

On comparison of mean prevalence of BPSD among subtypes of VCI, Apathy and Agitation were found to differ significantly in the assessed groups. Agitation was significantly more prevalent in CSVaD than in VaCIND (p=0.036), whereas the VaCIND group showed significantly less Apathy than both SVaD and CSVaD groups (p=0.025). Table 3 shows these results.

**Table 3:** Comparison of mean Neuropsychiatric Symptom prevalences among SVaD, CSVaD and VaCIND groups.

Neuropsychiatric symptoms	SVaD (n=810)	CSVaD (n=557)	VaCIND (n=159)	p-value	Post-hoc
Delusion	19.68 (10.68)	16.16 (8.78)	12.18 (9.09)	.519	–
Hallucination	10.79 (10.06)	12.21 (9.92)	1.85 (1.78)	.272	–
Agitation	33.45 (14.30)	41.33 (13.34)	16.30 (7.53)	.036	CSVaD≠VaCIND
Depression	52.63 (15.69)	53.34 (15.50)	45.94(23.40)	.798	–
Anxiety	36.37 (10.28)	36.09 (10.53)	31.28(10.61)	.759	–
Euphoria	5.41 (5.30)	9.99 (10.20)	3.04 (3.68)	.350	–
Apathy	49.02 (19.78)	50.24 (16.21)	17.14 (8.86)	.025	CSVaD≠VaCIND; SVaD≠VaCIND
Disinhibition	17.27 (9.11)	10.83 (11.11)	6.88 (1.76)	.254	–
Irritability	38.23 (12.21)	40.34 (19.32)	34.08 (8.73)	.839	–
Motor alterations	17.60 (14.28)	22.47 (17.23)	5.43 (7.73)	2.73	–
Sleeping alterations	33,47 (21,90)	44.85 (22.94)	26.13(28.83)	.447	–
Appetite alterations	37.98 (20.76)	40.23 (28.20)	26.82(14.83)	.710	–
Total	92.53 (4.55)	88.07 (16.42)	73.83(22.89)	.226	–

BPSD: Behavioral and Psychological Symptoms of Dementia; SVaD: Subcortical Vascular Dementia; CSVaD: Cortical-subcortical Vascular Dementia; VaCIND: Vascular Cognitive Impairment No-Dementia.

## DISCUSSION

The present study reviewed the characteristics of BPSD in VCI of different subtypes and clinical stages. The articles retrieved in this search showed that at least half of those individuals with VCI presented BPSD. Moreover, comparison between BPSD in SVaD and CSVaD revealed no differences in prevalence and profile of BPSD domains. Therefore, both subtypes of VaD are likely to present Depression and Apathy during the course of the disease. Other symptoms varied widely in prevalence among groups. As expected, VaCIND subjects had a lower prevalence of BPSD than VaD patients, a finding consistent with larger areas of spared brain tissue. Studies showed higher frequency of Delusions, Aberrant Motor Behavior and Sleep disorders in AD than in VaD subjects.^9,15,32,35^

Although AD was not part of the present search, available data on the differences between BPSD in vascular and neurodegenerative cognitive impairments were also examined. Studies that assessed AD also showed high prevalence of BPSD (up to 53.3%). More than half of the patients had at least one symptom, both in VaD and AD (AD 53.3% *vs* VaD 59.7%).^9^ In three studies, almost all patients with AD or VaD had some type of BPSD (AD=100% *vs* VaD=100%; AD=94.6% *vs* VaD=96.4%).^15,29,30^ Comparisons of BPSD prevalence in VaD and AD showed heterogeneous results. Depression and Apathy were more prevalent in VaD, whereas prevalence of the other symptoms varied widely across the studies. In another study, the prevalence for Depression was also higher in VCI (VCI=30% *vs* AD=15%).^31^ Several studies reported a higher prevalence of specific BPSD domains in AD than in VaD, as follows: Delusions (AD=22.4% *vs* VaD=8.1%),^9^ Aberrant Motor Behavior (AD=24.3% *vs* VaD=7.1%),^32^ Nocturnal Behavior/Sleep (AD=96.0% *vs* VaD=73.1%/AD=35.1% *vs* VaD=3.6%).^15,32^ These results indicate different patterns of behavioral symptoms between VCI and AD. However, since AD was not part of the search strategy for this review, further studies evaluating BPSD differences between VCI and AD are needed to allow more robust conclusions.

A number of issues in assessing BPSD may account for the disparity in results among studies. Neuropsychiatric Symptoms in dementia tend to fluctuate over time. Therefore, estimating prevalence of BPSD using a cross-sectional approach may not be appropriate. Moreover, since assessment of individual BPSD domains resulted in great variability across studies, identifying clusters of symptoms with similar underlying neurobiological correlates may allow better characterization of the behavioral profiles of VCI subtypes. In this regard, Frisoni et al. (1999)^36^ described a three-factor model of BPSD in AD: Mood (indicated by Anxiety, Apathy and Depression), Psychotic (Irritability/Lability, Delusions, Hallucinations and Agitation/Aggression), and Frontal Symptoms Domain (Euphoria and Disinhibition). Aalten et al. (2008)^37^ identified four neuropsychiatric subsyndromes: hyperactivity, psychosis, affective syndrome and apathy. Truzzi et al. (2013)^38^ compared BPSD clusters in subjects with dementia evaluated in two different countries: the Brazilian sample showed factors, such as Psychosis (Delusion, Hallucination, Euphoria, and Disinhibition), Mood (Depression, Anxiety, Agitation/Aggression, and Irritability), and Psychomotor (Apathy and Aberrant Motor Behavior), whereas factors drawn from a Norwegian sample were Psychosis (Delusion, Hallucination, and Aberrant Motor Behavior), Mood (Depression, Anxiety, Agitation/Aggression, Apathy and Irritability), and Frontal (Euphoria, Disinhibition, and Irritability).

Applying the cluster-model to the results of this review allows several conclusions to be drawn. Mood and psychomotor symptoms were more prevalent in patients with VCI whereas patients with AD may have more psychotic symptoms. Mood symptoms appeared to be equally prevalent in both SVaD and CSVaD.

Among the subtypes of VCI, data suggests that Apathy and Agitation were more prevalent in VaD than in VaCIND. Some differences between SVaD and CSVaD were identified. Agitation significantly differed in CSVaD as compared to VaCIND, but this difference was not found in relation to SVaD. This finding suggests a possible role of cortical function in the pathophysiology of this Neuropsychiatric Symptom. In fact, neurofibrillary tangles involving orbitofrontal cortex bilaterally have been previously associated with agitation among AD patients.^19^ Furthermore, both SVaD and CSVaD significantly differed from VaCIND in the mean prevalence of Apathy, which might indicate the importance of subcortical changes for the genesis of these symptoms. Apathy may be induced by changes in the neural networks generating and controlling goal-directed actions,^39^ which predominantly involve prefrontal cortex connections to basal ganglia, thalamus and limbic system structures.^20^ Thus, it seems plausible that disruption of the white-matter tracts between frontal cortex and basal ganglia by severe white-matter hyperintensities may result in Apathy. In addition, the severity of vascular load might also contribute to the occurrence of Apathy, as suggested by Chin et al. (2012),^40^ who found that patients with SVaD had higher scores for most individual items of the NPI than patients with SVaMCI, especially Apathy.

The present review has some limitations. Studies were included in which NPI was the only instrument evaluating BPSD. The inclusion of studies using other behavioral measurements, such as the Behave-AD, may have precluded direct comparisons among studies due to differences in the evaluated BPSD items evaluated. However, additional instruments would have provided a more overarching characterization of BPSD profiles in dementia subtypes. Moreover, since the NPI was not designed to assess BPSD in subjects with cognitive impairment below the dementia threshold, behavioral disturbances in VaCIND might have been underestimated. Secondly, some studies did not report the prevalence of each NPI item, which may have affected the results in this review. Finally, scoring on the NPI may be influenced by variables associated with the caregiver, such as burden. A version of the NPI which includes the clinician's impression of the patient's behavioral features (Neuropsychiatric Inventory-Clinician rating scale [NPI-C]) has been proposed and data has suggested that it may reduce bias associated with caregivers' imprecise information.^41^

Given the high prevalence, particularly of mood disorders, it is clear that a rigorous assessment of psychiatric features in VCI should be part of the routine examination of this patient group. Characterization of the behavioral profile of these subjects may allow a better comprehension of the disorder's pathophysiology and enable the development of more effective treatments for these conditions, positively impacting patient quality of life.
